# Impact of intensive treatment and remission on health-related quality of life in early and established rheumatoid arthritis

**DOI:** 10.1136/rmdopen-2016-000270

**Published:** 2016-08-26

**Authors:** I C Scott, F Ibrahim, C M Lewis, D L Scott, V Strand

**Affiliations:** 1 Academic Department of Rheumatology, Centre for Molecular and Cellular Biology of Inflammation, King's College London, London, UK; 2 Department of Medical and Molecular Genetics, King's College London, Guy's Hospital, London, UK; 3 Department of Rheumatology, Weston Education Centre, King's College Hospital, London, UK; 4 Division of Immunology/Rheumatology, Stanford University School of Medicine, Palo Alto, California, USA

**Keywords:** Rheumatoid Arthritis, DMARDs (synthetic), DMARDs (biologic), Disease Activity

## Abstract

**Objectives:**

To establish if using intensive treatment to reduce synovitis and attain remission in active rheumatoid arthritis (RA) improves all aspects of health-related quality of life (HRQoL).

**Methods:**

A secondary analysis of two randomised clinical trials (CARDERA and TACIT) was undertaken. CARDERA randomised 467 patients with early active RA to different disease-modifying antirheumatic drug (DMARD) regimens, including high-dose tapering corticosteroids. TACIT randomised 205 established patients with active RA to combination DMARDs (cDMARDs) or tumour necrosis factor-α inhibitors (TNFis). Short-Form 36 (SF-36) measured HRQoL across eight domains, generating physical (PCS) and mental (MCS) component summary scores. Linear regression evaluated 6-month intensive treatment impacts. Mean SF-36 scores, stratified by end point disease activity category, were compared with age/gender-matched population scores.

**Results:**

In CARDERA, intensive corticosteroid treatment gave significantly greater improvements in PCS but not MCS scores relative to placebo. In TACIT, all eight SF-36 domains had improvements from baseline exceeding minimal clinically important differences with cDMARDs and TNFis. Significantly greater improvements with TNFi relative to cDMARDs were reported in PCS only (p=0.034), after adjusting for covariates. Remission provided the best SF-36 profiles, but scores in physical functioning, role physical and general health in both trials remained below normative values. Patient global assessment of disease activity had a greater association with HRQoL than other disease activity score (DAS28) components.

**Conclusions:**

Intensive corticosteroid treatment in early RA improves physical but not mental health, relative to placebo. In established RA, cDMARDs and TNFi provide similar improvements in HRQoL. As remission optimises but fails to normalise HRQoL, a focus on treatment strategies targeting HRQoL is required.

**Trial registration numbers:**

CARDERA was registered as ISRCTN 32484878. TACIT was registered as ISRCTN 37438295; pre-results.

**Key messages**

**What is already known about this subject?**

- An important treatment goal in rheumatoid arthritis (RA) is optimising health-related quality of life (HRQoL).
- It is uncertain if intensive treatment and remission improve all aspects of HRQoL, with previous placebo-controlled trials of tumour necrosis factor-α inhibitors (TNFis) combined with methotrexate suggesting greater benefits on physical than mental health.

**What does this study add?**

- This secondary analysis of two randomised clinical trials shows that in early active RA, intensive corticosteroid therapy improves physical but not mental HRQoL relative to placebo, and in established active RA, intensive treatment with combination disease-modifying antirheumatic drugs and TNFis has similar impacts on HRQoL.
- It also demonstrates that in patients with previously active RA, attaining remission substantially improves, but fails to normalise, HRQoL (especially in established disease), and that the patient global assessment (PtGA) of disease activity has a strong association with HRQoL.

**How might this impact on clinical practice?**

- In order to optimise HRQoL in RA, it may be helpful to place an increased focus on improving PtGA scores; additional treatments based on reducing pain, fatigue and improving mood, which are likely to improve PtGA scores, may be of benefit in HRQoL.

## Introduction

Treating rheumatoid arthritis (RA) with disease-modifying antirheumatic drugs (DMARDs), biologics and corticosteroids is primarily intended to reduce synovitis. Efficacy is assessed by improving composite measures such as European League Against Rheumatism (EULAR) response criteria,^1^ ^2^ which mainly reflect reduced joint counts and acute phase markers. A key secondary goal is improving health-related quality of life (HRQoL). The treat-to-target (T2T) approach is based on the concept that HRQoL is maximised when synovitis is minimised through escalating drug treatment until patients achieve remission.^3^

HRQoL is a broad, multidimensional concept spanning physical and mental health, function, social support and socioeconomic status.^4^ Patients with RA in remission have substantially improved HRQoL compared with patients in higher disease activity states.^5^ However, intensive treatment and remission may not improve all aspects of HRQoL equally. Placebo-controlled trials evaluating treatment with tumour necrosis factor-α inhibitors (TNFis) combined with methotrexate suggest greater benefits for physical than mental health in DMARD-inadequate responders (DMARD-IRs),^6^ with no impact on mental health in methotrexate-naïve patients.^7^ ^8^ Focusing on minimising synovitis using intensive drug treatment risks overlooking aspects of HRQoL affected by pain, mood, anxiety, joint damage and deconditioning.

Our goal was to establish if using intensive treatment to reduce synovitis and attain remission improves all aspects of HRQoL. We undertook secondary analyses of two randomised clinical trials of intensive treatment in patients with early and established active RA. HRQoL was captured using the Short-Form 36 (SF-36). We had three aims: (1) establishing how intensive treatment with high-dose tapering corticosteroids, TNFi and combination DMARDs (cDMARDs) affects SF-36 domains; (2) evaluating the impact of remission and other disease activity score (DAS28)-defined disease activity categories on SF-36 domains and (3) evaluating which DAS28 components have the strongest associations with HRQoL.

## Methods

### Patients

We studied patients in the Combination Anti-Rheumatic Drugs in Early RA (CARDERA) and TNFi versus combination intensive therapy with conventional DMARDs in established RA (TACIT) trials; their primary results have been reported.^9^ ^10^

CARDERA recruited 467 patients with active early RA (<2 years duration) from 42 English centres. Patients were randomised to receive methotrexate alongside high-dose rapidly reducing corticosteroids,^11^ ciclosporin, placebo or both active treatments in a factorial design. Patients were assessed 6-monthly for 24 months.

TACIT recruited 205 patients with active established RA (>1 year duration) from 24 English centres. Patients were randomised to receive (a) TNFi (adalimumab, etanercept or infliximab), with a TNFi switch after 6 months if DAS28(ESR) reduction was <1.2; (b) intensive cDMARDs, with TNFi initiated after 6 months if DAS28(ESR) reduction was <1.2. Patients were assessed 6-monthly for 12 months.

### Health-related quality of life

#### Short-Form 36

SF-36 evaluated HRQoL in both trials. It measures HRQoL across eight domains: physical functioning (PF)—ability to perform physical activities; role physical (RP)—interference with work and other daily activities; bodily pain (BP); general health (GH)—how individuals evaluate their health; vitality (VT)—fatigue, pep and energy; social functioning (SF)—interference with social activities; role emotional (RE)—interference with work/daily activities due to emotional problems; general mental health (MH)—nervousness and depression.^12^ These domains are scored 0–100; higher scores indicate better health. Domain scores can be normalised, z-transformed and combined into physical and mental component summary scores (PCS and MCS) providing summary measures of physical and mental health, relative to population means of 50 (SD 10).^13^ The PCS positively weights the physical domains and VT and negatively weights the other mental domains; the MCS positively weights the four mental domains and negatively weights the four physical domains. Weights used are factor score coefficients derived from the general population.^13^ Minimal clinically important differences (MCIDs) in SF-36 domain and component summary scores are 5.0 and 2.5 units, respectively.^14^

#### Short-Form 6D

SF-36 scores were converted into the Short-Form 6D (SF-6D), a health utility measure ranging from 0.290 (worst health) to 1.000 (perfect health), using the Ara and Brazier algorithm.^15^ ^16^ Its minimum important difference (MID) is 0.041.^17^

### Analysis time point

We evaluated treatment effects on HRQoL over the first 6 months in CARDERA and TACIT for two reasons. First, the COBRA corticosteroid regimen in CARDERA (initially 60 mg/day, tapered to 7.5 mg/day by weeks 7–28 and stopped by week 36)^11^ had maximal effects at 6 months. Second, in TACIT after 6-month cDMARD treatment, 41% of patients switched to TNFis; 6-month results specifically compared TNFi with cDMARDs. To ensure 6 months was not too early a time point to observe treatment effects on HRQoL, we undertook a sensitivity analysis looking at treatment effects on HRQoL over 12 months (see online supplementary table 1).

The impact of remission and DAS28 components on HRQoL was assessed at study end points (24 months in CARDERA; 12 months in TACIT) when remission rates were greatest.

### Statistical analysis

#### Impact of treatment on SF-36

Treatment effects were evaluated using linear regression models including the 6-month changes in each SF-36 domain and summary score as the response variable. An unadjusted model included treatment (active vs placebo corticosteroids in CARDERA; TNFi vs cDMARD therapy in TACIT) as the explanatory variable. An adjusted model included treatment, baseline SF-36 domain/summary score, age, sex and disease duration as explanatory variables.

#### Impact of remission and disease activity categories on SF-36

Mean SF-36 domain scores at the final time point in CARDERA and TACIT were plotted on spydergrams stratified by (a) DAS28(ESR) activity category: remission (DAS28(ESR) <2.6), low disease activity (LDA; ≥2.6 to <3.2), moderate disease activity (MDA; 3.2–5.1), high disease activity (HDA; >5.1) and (b) remission versus non-remission according to each DAS28 component: tender joint count (TJC) ≤1, swollen joint count (SJC) ≤1, patient global assessment (PtGA) of disease activity on a 100 mm visual analogue scale ≤10, erythrocyte sedimentation rate (ESR) ≤20 mm/hour. These component cut-offs represent the preliminary American College of Rheumatology (ACR)/EULAR Boolean-based definition of RA remission for clinical trials.^18^ As C reactive protein (CRP) data were not available, a normal ESR level was considered indicative of acute phase response remission.

#### Associations between DAS28 components and SF-36 summary scores

To minimise type I error from multiple testing (4 DAS28 components; 8 health domains), associations between DAS28(ESR) components and PCS and MCS were tested. Linear regression models used final time point PCS and MCS scores as response variables, and SJC, TJC, ESR and PtGA as explanatory variables, adjusted for covariates (treatment, age, sex and disease duration). Model 1 tested each DAS28(ESR) component separately. Model 2 included all DAS28(ESR) components as explanatory variables. To ensure multicollinearity between DAS28(ESR) components was not an issue in model 2, variance inflation factors (VIFs) were calculated for each predictor; VIF was <2 for all explanatory variables.^19^ Standardised β values were calculated enabling direct comparison of effect sizes of each DAS28(ESR) component on PCS and MCS.

#### Missing data imputation

In CARDERA, missing data had been imputed at all time points using last observation carried forward (LOCF) analysis.^10^ Missing data were imputed in 19% of patients at 24 months; an observed case analysis had excluded a significant impact of the LOCF assumption on the study end points, which included PCS and MCS scores. For consistency across studies, we imputed missing TACIT data using LOCF (undertaken at 6 months in 5 and 18 patients for SF-36 domain scores and DAS28(ESR) components, respectively, and at 12 months in 15 and 16 patients for SF-36 domain scores and DAS28(ESR) components, respectively). We undertook an additional analysis using non-imputed TACIT data to ensure our findings were not biased by LOCF imputation (see online supplementary tables 2 and 3).

10.1136/rmdopen-2016-000270.supp1 Supplementary tables

#### Normative SF-36 profiles

Age-matched and gender-matched US normative scores (A/G norms) were generated for CARDERA and TACIT protocol populations using data published in SF-36 manuals and updates.^20^ It was not possible to use UK A/G norms as these data are not publicly available, although existing studies have highlighted similarities in mean SF-36 domain scores between UK and US populations.^21^ ^22^

#### Statistical software

Analyses were performed in R (V.3.1.3).

### Ethics, consent and permissions

CARDERA (ISRCTN 32484878 and Research Ethics Committee (REC) reference: MREC (1) 99/04) and TACIT (ISRCTN 37438295 and REC reference: MREC Ref 07/Q0505/57) were approved by research ethics committees. All patients provided informed written consent.

## Results

### Baseline characteristics

In both trials, most patients were female, with a mean age in the sixth decade (table 1). As expected from the inclusion criteria for each trial, mean disease duration in TACIT (8.2 years) was higher than CARDERA (0.3 years). TACIT patients also had higher baseline DAS28(ESR) (mean 6.26) compared with CARDERA (mean 5.78), attributable to higher TJC and PtGA scores. Baseline health assessment questionnaire (HAQ) levels were slightly higher in TACIT (mean 1.85) than CARDERA (mean 1.59). PCS scores were lower in TACIT (mean 26.0) than CARDERA (mean 29.6); MCS scores were similar in both trials (CARDERA mean 39.8; TACIT mean 39.2).

**Table 1:** Patient baseline characteristics

	CARDERA (n=467)			TACIT (n=205)		
Characteristic	Placebo (n=236)	Steroids (n=231)	p Value	cDMARDs (n=104)	TNFi (n=101)	p Value
Demographic						
Number (%) female	157 (67)	166 (72)	0.251	73 (70)	79 (78)	0.249
Age (years)	53.8 (13.6)	54.5 (11.5)	0.567	58.0 (12.9)	56.7 (11.0)	0.459
RA specific						
Disease duration (years)	0.3 (0.4)	0.4 (0.5)	0.082	7.3 (8.4)	9.2 (9.2)	0.126
DAS28	5.84 (1.27)	5.71 (1.30)	0.266	6.21 (0.92)	6.30 (0.81)	0.461
SJC	10.0 (6.5)	9.7 (6.1)	0.577	10.5 (6.1)	10.8 (6.7)	0.753
ESR	40.9 (28.9)	40.8 (29.6)	0.979	33.1 (26.1)	30.1 (22.8)	0.379
TJC	12.3 (7.4)	11.3 (7.8)	0.130	16.4 (7.1)	17.5 (6.7)	0.259
PtGA	54.3 (26.7)	55.6 (26.0)	0.582	68.1 (19.7)	68.2 (21.3)	0.988
HAQ	1.59 (0.68)	1.59 (0.69)	0.984	1.80 (0.59)	1.90 (0.67)	0.251
HRQoL						
PF	33.6 (24.5)	34.3 (24.0)	0.754	30.1 (22.6)	24.6 (20.9)	0.067
RP	14.4 (29.5)	17.9 (32.8)	0.233	14.9 (30.1)	12.4 (26.1)	0.521
BP	33.2 (21.0)	34.4 (21.4)	0.541	28.1 (16.3)	26.3 (17.8)	0.435
GH	44.4 (21.1)	46.4 (19.9)	0.305	35.8 (18.2)	31.4 (16.8)	0.074
VT	31.2 (20.3)	34.7 (20.7)	0.063	30.3 (21.4)	26.6 (19.0)	0.185
SF	48.3 (31.2)	54.4 (29.7)	0.030	50.2 (25.2)	42.1 (25.3)	0.022
RE	38.3 (43.7)	46.5 (44.9)	0.046	43.9 (44.9)	35.3 (44.9)	0.172
MH	59.5 (20.0)	61.6 (19.0)	0.252	61.9 (20.2)	58.8 (23.1)	0.305
PCS	29.6 (8.5)	29.6 (9.5)	0.969	26.5 (7.5)	25.5 (7.8)	0.356
MCS	38.3 (15.2)	41.3 (14.3)	0.025	40.8 (14.8)	37.6 (14.6)	0.120

Data given as mean (SD) unless otherwise stated; p values for continuous and categorical variables derived from t-tests and χ^2^ tests, respectively.

BP, bodily pain; cDMARDs, combination disease-modifying antirheumatic drugs; DAS28, disease activity score; ESR, erythrocyte sedimentation rate; GH, general health; HRQoL, health-related quality of life; MCS, mental component summary; MH, mental health; PCS, physical component summary; PF, physical functioning; PtGA, patient global assessment of disease activity; RA, rheumatoid arthritis; RE, role emotional; RP, role physical; SF, social functioning; SJC, swollen joint count; TJC, tender joint count; TNFi, tumour necrosis factor-α inhibitor; VT, vitality.

Within-trial comparisons of baseline patient characteristics between treatment arms showed patients were well matched (table 1). Significant differences were observed between treatment arms for some SF-36 domains: in CARDERA, SF, RE and MCS scores were significantly higher in the active steroid group; in TACIT, SF was significantly lower in the TNFi group.

### Impact of corticosteroids versus placebo on SF-36 domains

**Figure 1:**
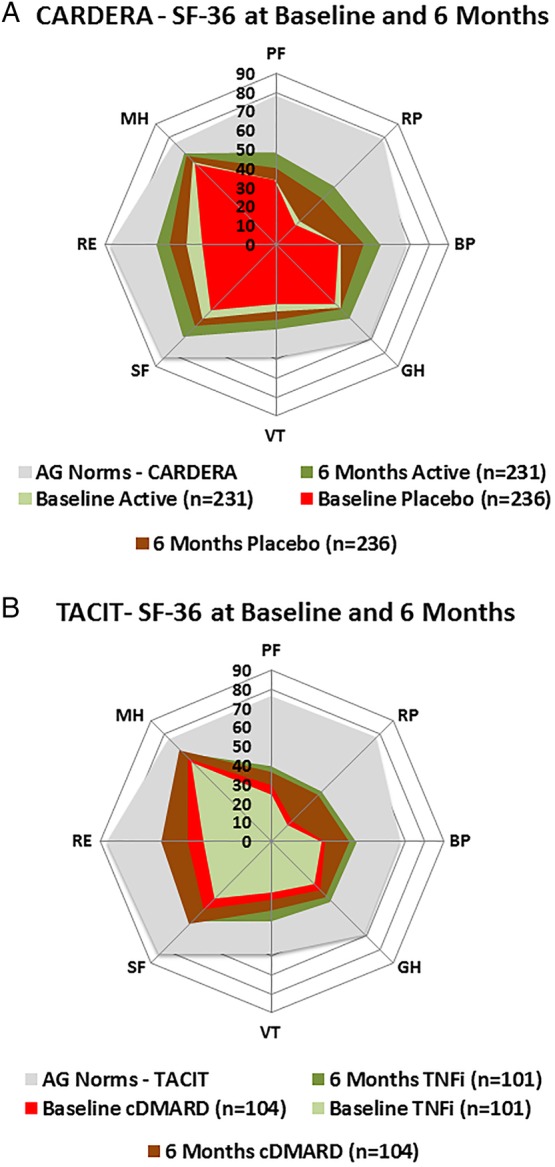
Mean Short-Form 36 (SF-36) domain scores at baseline and 6 months in patients receiving active or placebo corticosteroids, cDMARDs or TNFis. (A) Patients with early RA in the CARDERA trial randomised to receive either active or placebo high-dose tapering corticosteroids (baseline placebo and 6 months placebo=mean SF-36 domain scores at 0 and 6 months in patients receiving placebo corticosteroids; baseline active and 6 months active=mean SF-36 domain scores at 0 and 6 months in patients receiving active corticosteroids; A/G norms=mean SF-36 domain scores in an age-matched and gender-matched normative US population). (B) Patients with established RA in the TACIT trial randomised to receive either cDMARD or TNFi (baseline cDMARD and 6 months cDMARD=mean SF-36 domain scores at 0 and 6 months in patients receiving cDMARDs; baseline TNFi and 6 months TNFi=mean SF-36 domain scores at 0 and 6 months in patients receiving TNFi; A/G norms=mean SF-36 domain scores in an age-matched and gender-matched normative US population). BP, bodily pain; cDMARD, combination disease-modifying antirheumatic drug; GH, general health; MH, mental health; PF, physical functioning; RA, rheumatoid arthritis; RE, role emotional; RP, role physical; SF, social functioning; TNFi, tumour necrosis factor-α inhibitor; VT, vitality.

In CARDERA, 6-month increases in mean scores exceeding MCID were reported in all eight SF-36 domains with active and placebo corticosteroids, with the exception of GH in the placebo arm (figure 1). Active corticosteroids resulted in significant improvements in all physical health domains, relative to placebo (table 2). Over 6-months increases in PF, RP, BP, GH and PCS scores were estimated to be 7.97 (p<0.001), 7.34 (p=0.047), 8.14 (p<0.001), 5.00 (p=0.003) and 3.78 (p<0.001) units greater with corticosteroids than placebo, respectively, when evaluated using the adjusted linear regression model. In contrast, corticosteroids had no effect on any mental health domains when evaluated using unadjusted and adjusted linear regression models. Despite improving physical health, 6-month SF-36 profiles remained substantially lower than those of A/G norms (figure 1). A sensitivity analysis evaluating the impact of corticosteroids on changes in SF-36 domain scores over 12 months showed no treatment effect of corticosteroids compared with placebo, suggesting the benefits of high-dose tapering corticosteroids on physical HRQoL are not sustained over time (see online supplementary table S1).

**Table 2:** Effect of intensive treatment on 6-month changes in SF-36 domains

SF-36 domain	CARDERA				TACIT			
	Model 1: Unadjusted		Model 2: Adjusted		Model 1: Unadjusted		Model 2: Adjusted	
	β (SE)	p Value	β (SE)	p Value	β (SE)	p Value	β (SE)	p Value
PF	7.55 (2.27)	<0.001	7.97 (2.18)	<0.001	8.61 (3.83)	0.026	5.43 (3.62)	0.135
RP	5.52 (3.94)	0.162	7.34 (3.69)	0.047	5.05 (6.28)	0.423	1.55 (5.78)	0.789
BP	7.59 (2.34)	0.001	8.14 (2.20)	<0.001	5.93 (3.29)	0.073	3.97 (2.89)	0.170
GH	4.52 (1.78)	0.011	5.00 (1.68)	0.003	7.38 (2.97)	0.014	4.16 (2.64)	0.117
VT	1.39 (1.86)	0.454	2.60 (1.76)	0.140	7.46 (3.28)	0.024	4.72 (2.91)	0.107
SF	1.86 (2.69)	0.488	4.57 (2.42)	0.060	6.01 (3.94)	0.129	0.11 (3.47)	0.975
RE	−1.51 (4.52)	0.739	4.36 (3.82)	0.254	1.72 (7.72)	0.824	−6.93 (6.25)	0.269
MH	−0.23 (1.77)	0.898	0.66 (1.61)	0.684	1.09 (3.45)	0.753	−2.25 (2.76)	0.415
PCS	3.84 (1.02)	<0.001	3.78 (0.97)	<0.001	3.70 (1.51)	0.015	3.04 (1.42)	0.034
MCS	−1.33 (1.32)	0.316	0.36 (1.16)	0.757	0.85 (2.29)	0.710	−1.99 (1.91)	0.301

Adjusted model includes following covariates: age, gender, disease duration and baseline SF-36 domain/summary score; cDMARD used as treatment reference group in TACIT analysis.

BP, bodily pain; cDMARD, combination disease-modifying antirheumatic drug; GH, general health; MCS, mental component summary; MH, mental health; PCS, physical component summary; PF, physical functioning; RE, role emotional; RP, role physical; SF, social functioning; SF-36, Short-Form 36; VT, vitality.

### Impact of TNFi versus cDMARDs on SF-36 domains

In TACIT, 6-month increases in mean scores exceeding MCID were reported in all eight SF-36 domains with cDMARDs and TNFi (figure 1). A total of 6 months of TNFi therapy provided a significantly greater improvement in PCS compared with cDMARD therapy (table 2); the increase in PCS was estimated to be 3.04 units greater (p=0.034) with TNFi compared with cDMARDs when evaluated using the adjusted model. Significantly greater improvements were reported in PF, GH and VT in the unadjusted model, although these domains lost significance after adjusting for their baseline scores and other covariates. As in CARDERA, despite TNFi and cDMARD therapy improving SF-36 domains, HRQoL remained substantially below that of A/G norms in both treatment arms (figure 1). A sensitivity analysis evaluating the impact of TNFi versus cDMARDs on changes in SF-36 domain scores over 12 months showed no effect of TNFi versus cDMARDs (see online supplementary table S1).

### Impact of remission and disease activity categories on SF-36 domains

#### SF-36 domains by DAS28 category

**Figure 2:**
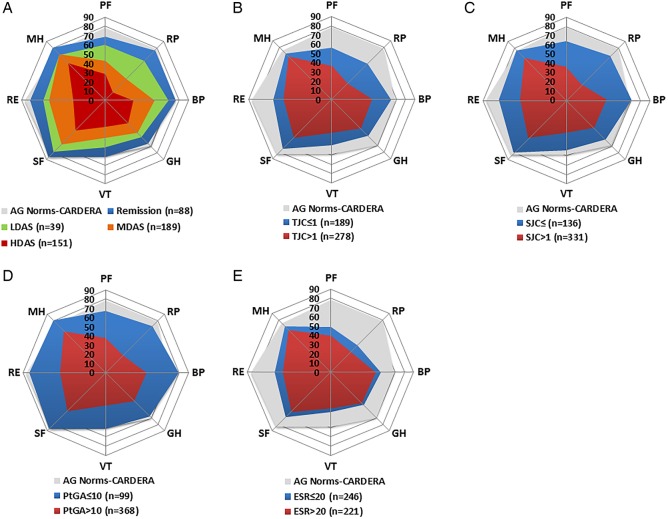
Mean Short-Form 36 (SF-36) domain scores stratified by DAS28-defined disease activity category, and DAS28-component defined remission in CARDERA. (A) Mean SF-36 domain scores in patients in remission (DAS28<2.6), low disease activity (LDA; ≥2.6–<3.2), moderate disease activity (MDA; 3.2–5.1) and high disease activity (HDA; >5.1). (B) Mean scores in patients meeting/not meeting tender joint count (TJC)-defined remission (TJC≤1). (C) Mean scores in patients meeting/not meeting swollen joint count (SJC)-defined remission (SJC≤1). (D) Mean scores in patients meeting/not meeting patient global assessment (PtGA) of disease activity-defined remission (100 mm PtGA≤10). (E) Mean scores in patients meeting/not meeting erythrocyte sedimentation rate (ESR)-defined remission (ESR≤20 mm/hour). These DAS28-component cut-offs represent the preliminary American College of Rheumatology (ACR)/European League Against Rheumatism (EULAR) Boolean-based definition of rheumatoid arthritis (RA) remission for clinical trials. A/G norms=mean SF-36 domain scores in age-matched and gender-matched individuals from the normative US population. Space between grid lines is 10 units, which represents twice minimal clinically important differences (MCIDs). BP, bodily pain; GH, general health; MH, mental health; PF, physical functioning; RE, role emotional; RP, role physical; SF, social functioning; VT, vitality.

**Figure 3:**
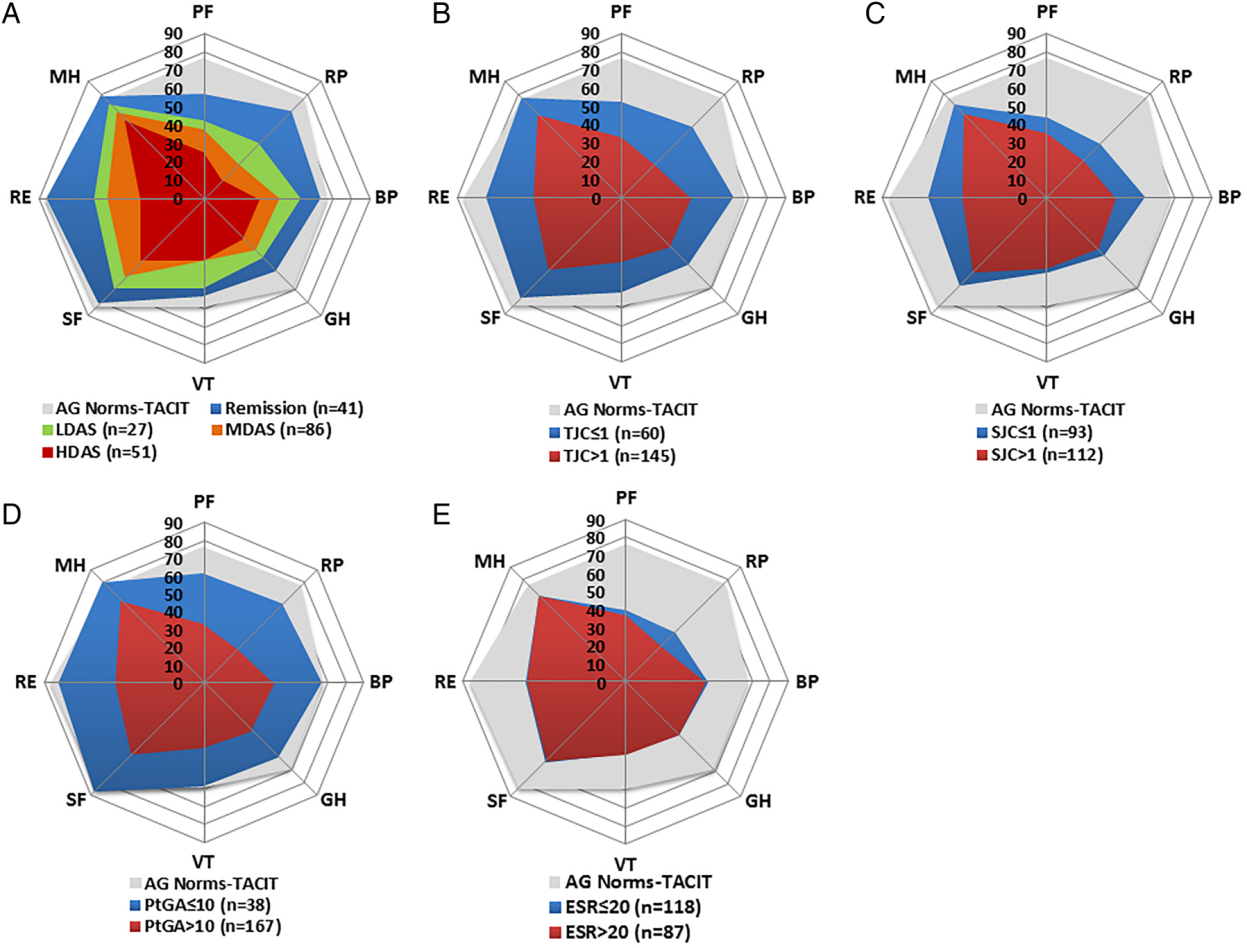
Mean Short-Form 36 (SF-36) domain scores stratified by DAS28-defined disease activity category, and DAS28-component defined remission in TACIT. (A) Mean SF-36 domain scores in patients in remission (DAS28<2.6), low disease activity (LDA; ≥2.6–<3.2), moderate disease activity (MDA; 3.2–5.1) and high disease activity (HDA; >5.1). (B) Mean scores in patients meeting/not meeting tender joint count (TJC)-defined remission (TJC≤1). (C) Mean scores in patients meeting/not meeting swollen joint count (SJC)-defined remission (SJC≤1). (D) Mean scores in patients meeting/not meeting patient global assessment (PtGA) of disease activity-defined remission (100 mm PtGA≤10). (E) Mean scores in patients meeting/not meeting erythrocyte sedimentation rate (ESR)-defined remission (ESR≤20 mm/hour). These DAS28-component cut-offs represent the preliminary American College of Rheumatology (ACR)/European League Against Rheumatism (EULAR) Boolean-based definition of rheumatoid arthritis (RA) remission for clinical trials. A/G norms=mean SF-36 domain scores in an age-matched and gender-matched normative US population. Space between grid lines is 10 units, which represents twice minimal clinically important differences (MCIDs). BP, bodily pain; GH, general health; MH, mental health; PF, physical functioning; RE, role emotional; RP, role physical; SF, social functioning; VT, vitality.

In both trials, increases in all SF-36 domains were observed with reducing DAS28(ESR) disease activity categories (figures 2 and 3). Remission provided the highest mean SF-36 domain scores in CARDERA and TACIT.

In CARDERA (figure 2), patients in remission reported mean scores 9.6, 2.9, 3.9 and 5.0 units lower than A/G norms for PF, RP, GH and RE, respectively. Mean scores in BP, VT, SF and MH were 7.9, 1.6, 3.1 and 4.8 units higher, respectively.

In TACIT (figure 3), patients in remission reported mean scores 19.6, 9.8, 3.3, 13.3, 5.7, 3.1 and 0.7 units lower than A/G norms for PF, RP, BP, GH, VT, SF and RE, respectively. Mean scores in MH were 2.7 units higher.

#### SF-36 domains by DAS28 component remission

In both CARDERA and TACIT, PtGA-defined remission resulted in the highest mean domain scores and ESR-defined remission resulted in the lowest mean domain scores, compared with remission defined by other DAS28(ESR) components (figures 2 and 3).

### Association between DAS28 components and SF-36 summary scores

In CARDERA, all four DAS28(ESR) components had significant associations with PCS and MCS scores at 24 months in both models (model 1 testing each component separately; model 2 testing all components in same model) except TJC, which did not have a significant association with PCS in model 2 (p=0.977; table 3). PtGA had the largest effect on PCS (model 1 standardised β=−0.57) and MCS (model 1 standardised β=−0.44) scores.

**Table 3:** Associations between DAS28 components and SF-36 PCS and MCS at final trial time points

DAS28 component	CARDERA				TACIT			
	Model 1: DAS28 components tested individually		Model 2: DAS28 components tested in same model		Model 1: DAS28 components tested individually		Model 2: DAS28 components tested in same model	
	Standardised β (SE)	p Value	Standardised β (SE)	p Value	Standardised β (SE)	p Value	Standardised β (SE)	p Value
PCS								
SJC	−0.45 (0.04)	<0.001	−0.19 (0.05)	<0.001	−0.24 (0.07)	<0.001	−0.06 (0.08)	0.412
TJC	−0.33 (0.04)	<0.001	0.00 (0.05)	0.977	−0.33 (0.07)	<0.001	−0.08 (0.09)	0.370
ESR	−0.13 (0.05)	0.005	−0.08 (0.04)	0.036	−0.02 (0.07)	0.804	0.03 (0.07)	0.676
PtGA	−0.57 (0.04)	<0.001	−0.45 (0.05)	<0.001	−0.43 (0.06)	<0.001	−0.36 (0.08)	<0.001
MCS								
SJC	−0.29 (0.04)	<0.001	−0.12 (0.06)	0.029	−0.16 (0.07)	0.024	0.05 (0.08)	0.527
TJC	−0.13 (0.05)	0.004	0.15 (0.05)	0.003	−0.34 (0.07)	<0.001	−0.16 (0.09)	0.080
ESR	−0.14 (0.05)	0.004	−0.12 (0.04)	0.008	−0.04 (0.07)	0.578	0.00 (0.07)	0.974
PtGA	−0.44 (0.04)	<0.001	−0.43 (0.05)	<0.001	−0.41 (0.06)	<0.001	−0.33 (0.08)	<0.001

All linear regression models include age, gender, disease duration and treatment as covariates; CARDERA model includes the 24-month PCS and MCS as the response variables; TACIT model includes the 12-month PCS and MCS as the response variables; the VIFs were <2 for all explanatory variables in model 2. DAS28, disease activity score; ESR, erythrocyte sedimentation rate; MCS, mental component summary; PCS, physical component summary; PtGA, patient global assessment of disease activity; SF-36, Short-Form 36; SJC, swollen joint count; TJC, tender joint count.

In TACIT, SJC, TJC and PtGA, but not ESR had significant associations with PCS and MCS scores in model 1 (table 3); as in CARDERA, the largest effect size was observed with PtGA (PCS and MCS model 1 standardised β values of −0.43 and −0.41, respectively). Only PtGA retained a significant association with PCS (p<0.001) and MCS (p<0.001) scores in model 2.

### SF-6D utility scores

SF-6D scores improved in CARDERA and TACIT with all treatments. In CARDERA, SF-6D scores increased from 0.594 to 0.669 (change 0.076) over 6 months with active corticosteroids, and from 0.577 to 0.633 (change 0.057) with placebo. In TACIT, SF-6D scores increased from 0.548 to 0.629 (change 0.081) over 6 months with TNFi and 0.574 to 0.626 (change 0.052) with cDMARDs. Improvements in both treatment arms in both trials exceeded MID.

## Discussion

We have undertaken secondary analyses of two completed randomised clinical trials in early and established RA. Our first aim was to evaluate the effect of intensive treatments—high-dose corticosteroids versus placebo in early RA, and cDMARDs versus TNFis in established RA—on SF-36 domains during the first 6 months of treatment. With all active treatments, there were improvements in all eight SF-36 domains, which exceeded MCIDs (5 units). However, in early RA, corticosteroids only provided improvements beyond placebo in physical but not mental SF-36 domains. Furthermore, in established RA, there were similar improvements with cDMARDs and TNFis. These results show that in these two trials intensive treatments resulted in limited additional benefits on HRQoL compared with less intensive treatments.

Our second aim was to evaluate the impact of remission and other DAS28-defined disease activity categories on SF-36 domains at trial end points. Patients in remission had better HRQoL profiles than patients in higher disease activity states. However, scores in PF, RP and GH in both trials remained below normative values, with large deficits observed in established RA patients enrolled to TACIT. The IMPROVED trial suggested that in patients with early inflammatory arthritis, attaining prompt remission normalises HRQoL.^23^ Overall, these findings suggest that in patients with established RA and in some patients with early RA, attaining remission does not normalise HRQoL, but attaining early remission in patients with early inflammatory arthritis may be sufficient to achieve this goal.

Our third aim was to assess which DAS28 components had the strongest associations with HRQoL at trial end points. In CARDERA and TACIT, PtGA had the strongest association with PCS and MCS scores; associations with SJC and ESR levels were weaker. Although different types of patient-reported outcomes such as PtGA and SF-36 component summary scores may be expected to be inter-related, our finding that PtGA was most strongly associated with physical and mental HRQoL compared with other DAS28 components is of clinical importance. It suggests that in order to optimise HRQoL in RA, it might be helpful to place an increased focus on improving PtGA scores. In routine practice, the PtGA is not a key driver of rheumatologists' treatment decisions, with Dutch^24^ and Canadian^25^ studies of RA patients demonstrating that SJC and physician global assessment of disease activity to be central drivers of treatment escalation. As PtGA does not purely reflect how active a patient considers their disease to be—a previous study by Studenic *et al*^26^ showed that 76% and 1.3% of the variability in PtGA was explained by pain and physical function, respectively—treatment intensity decisions should not be based on PtGA alone. However, there is likely to be a role for more holistic approaches to managing RA patients that move beyond purely targeting remission, and consider treating other disease impacts such as pain, fatigue and mood, which are likely to improve PtGA scores.

The failure of TNFi to improve individual SF-36 domains beyond cDMARDs in TACIT was of interest. Although limited by a modest sample size, TACIT provided only little evidence that 6-months TNFi with DMARDs was superior to cDMARDs at improving HRQoL. Previous trials of TNFi with methotrexate versus methotrexate monotherapy in DMARD-IR patients show significant improvements in PCS and MCS scores with biologics, although the effects are greater on physical than mental health.^6^ To our knowledge, only one other study has compared the effect of TNFi and cDMARDs on HRQoL. A secondary analysis of the BeST study showed 12-month EuroQol scores were similar with sequential DMARD monotherapy, step-up combination DMARDs, initial combination DMARDs with high-dose tapering prednisolone, and methotrexate with infliximab, although prompter improvements were seen in the latter two groups.^27^ TACIT and BeST suggest TNFis have only limited benefits on HRQoL compared with cDMARDs. There is insufficient evidence, however, to know if this is correct in all clinical situations; further large trials are needed to confirm the relative benefits of different intensive treatment regimens.

In CARDERA and TACIT SF-36, health profiles were superior in patients attaining remission, compared with those attaining LDA. This replicates previous work by Radner *et al*,^28^ who reported that in 356 RA patients with longstanding RA from Austria, remission gave superior SF-36 profiles to LDA. Interestingly, as in our analysis, Radner *et al* also found most SF-36 domains in RA patients in remission were below general population levels. Their findings therefore support our view that additional management strategies are needed to normalise HRQoL in addition to attaining remission.

Our study has several strengths. As a secondary analysis of two clinical trials, assessments were standardised. Its main findings were similar in early and established RA patients receiving different intensive treatments. Patients were recruited from many English centres, which followed consistent healthcare approaches. It also has several limitations. As a post-hoc analysis, it did not test a prespecified hypothesis. It was restricted to assessing treatment impacts in active RA, limiting its generalisability to all RA populations. It focused on 6-month and 12-month HRQoL changes; longer time frames could show greater benefits. It used a generic HRQoL assessment (SF-36); disease-specific measures like RAQoL^29^ may better capture treatment effects. TACIT was a non-inferiority trial comparing cDMARDs with TNFi strategies; our analysis was underpowered to detect small improvements in SF-36.^9^ Finally, short-term high-dose steroids are not widely used in current practice.

Our key finding that physical HRQoL remained impaired even when intensive treatment achieved remission could reflect several underlying drivers. Loss of lean muscle mass, which is linked with RA disability,^30^ may occur rapidly in active RA and will not be reversed with drug treatment. Joint damage, though less common in contemporary RA cohorts, has detrimental effects on physical function^31^ and cannot be reversed by drug treatment. Persistent pain may also impair physical health. Finally, clinical remission may be insensitive at detecting low-level synovitis and inflammation, which could affect physical HRQoL.

Patient surveys suggest current RA management does not fully address their needs and expectations.^32^ ^33^ The ‘RAISE patient needs’ survey found few patient–physician consultations discussed HRQoL.^32^ The ‘Good Days Fast’ and ‘Getting to Your Destination Faster’ surveys found most patients rated ‘having a good day’ as their preferred target for RA management; being free of fatigue and pain often characterised ‘good days’.^33^ In these surveys, pain was a prevalent problem for patients with RA. Our findings suggest that attaining remission, though crucial to improving RA outcomes, is insufficient by itself to entirely normalise HRQoL in active RA, particularly in established disease. New ways are needed to identify and treat specifically impaired areas of HRQoL—including pain and fatigue—as an adjunct to treatments that reduce synovitis assessed by DAS28. Potential options include using psychological approaches, increasing exercise and improving pain management.
