# Supplementary material for: Impact of intensive treatment and remission on health-related quality of life in early and established rheumatoid arthritis
Source: RMD Open. 2016 Aug 26;2(2):e000270. doi: 10.1136/rmdopen-2016-000270 (PMC5013499; doi:10.1136/rmdopen-2016-000270)
Supplement: Supplementary tables [file rmdopen-2016-000270supp_tables.pdf]

THE IMPACT OF INTENSIVE TREATMENT AND REMISSION ON HEALTH-RELATED QUALITY OF LIFE IN EARLY AND ESTABLISHED RHEUMATOID ARTHRITIS

Scott IC, Fowzia I, Lewis CM, Scott DL, Strand V

SUPPLEMENTARY DATA

Supplementary Table 1- Effect of Intensive Treatment on 12-month Changes in SF-36 Domains

SF-36 Domain	CARDERA				TACIT			
	Model 1- Unadjusted		Model 2- Adjusted		Model 1- Unadjusted		Model 2- Adjusted	
	β (SE)	*P-value*	*β (SE)*	*P-value*	*β (SE)*	*P-value*	*β (SE)*	*P-value*
PF	3.00 (2.27)	0.187	3.42 (2.17)	0.116	1.66 (3.85)	0.668	-2.26 (3.63)	0.534
RP	1.12 (3.75)	0.766	3.19 (3.52)	0.366	0.36 (6.13)	0.953	-1.71 (5.75)	0.767
BP	0.03 (2.33)	0.989	1.07 (2.16)	0.622	-0.18 (3.64)	0.960	-2.72 (3.17)	0.392
GH	0.42 (1.88)	0.822	1.17 (1.74)	0.504	-0.91 (3.15)	0.773	-3.88 (2.90)	0.182
VT	-1.24 (2.06)	0.548	0.68 (1.89)	0.719	3.16 (3.31)	0.340	1.28 (3.05)	0.675
SF	-1.85 (2.81)	0.511	1.65 (2.46)	0.501	2.55 (4.28)	0.552	-4.06 (3.68)	0.270
RE	-1.47 (4.70)	0.755	4.53 (3.96)	0.253	-0.19 (7.78)	0.980	-8.25 (6.28)	0.190
MH	-2.15 (1.81)	0.234	-1.05 (1.65)	0.524	0.27 (3.40)	0.937	-2.84 (2.72)	0.299
PCS	1.13 (0.99)	0.254	1.18 (0.95)	0.211	0.17 (1.63)	0.917	-0.72 (1.48)	0.626
MCS	-1.60 (1.44)	0.268	0.36 (1.24)	0.771	0.34 (2.29)	0.884	-2.37 (1.86)	0.204

*Adjusted model includes following covariates: age, gender, disease duration and baseline SF-36 domain/summary score; cDMARD used as treatment reference group in TACIT analysis.

Supplementary Table 2- Effect of Intensive Treatment on 6-month Changes in SF-36 Domains in TACIT Using Non-Imputed Data

	**Model 1- Unadjusted**		**Model 2- Adjusted**	
**SF-36 Domain**	*β (SE)*	*P-value*	*β (SE)*	*P-value*
PF	8.50 (3.93)	0.032	5.44 (3.69)	0.142
RP	4.50 (6.42)	0.484	0.87 (5.89)	0.883
BP	5.60 (3.36)	0.097	4.08 (2.94)	0.166
GH	7.30 (3.03)	0.017	4.14 (2.70)	0.126
VT	7.35 (3.36)	0.030	4.65 (2.97)	0.119
SF	5.75 (4.03)	0.155	-0.26 (3.54)	0.941
RE	1.33 (7.91)	0.866	-7.30 (6.37)	0.253
MH	0.92 (3.54)	0.795	-2.53 (2.82)	0.371
PCS	3.61 (1.54)	0.020	3.01 (1.45)	0.039
MCS	0.73 (2.34)	0.754	-2.12 (1.96)	0.279

*Adjusted model includes following covariates: age, gender, disease duration and baseline SF-36 domain/summary score; cDMARD used as treatment reference group.

Supplementary Table 3 - Associations between DAS28 Components and SF-36 PCS and MCS at Final Time-Point in TACIT Using Non-Imputed Data

	**Model 1- DAS28 Components Tested Individually**		**Model 2- DAS28 Components Tested In Same Model**	
**DAS28-Component**	*Standardised β (SE)*	*P-value*	*Standardised β (SE)*	*P-value*
		**PCS**		
SJC	-0.22 (0.07)	0.003	-0.05 (0.08)	0.547
TJC	-0.35 (0.07)	<0.001	-0.13 (0.10)	0.187
ESR	-0.02 (0.07)	0.775	0.03 (0.07)	0.673
PGA	-0.44 (0.07)	<0.001	-0.34 (0.09)	<0.001
		**MCS**		
SJC	-0.10 (0.07)	0.171	0.09 (0.08)	0.275
TJC	-0.31 (0.07)	<0.001	-0.15 (0.10)	0.116
ESR	-0.06 (0.07)	0.418	-0.03 (0.07)	0.657
PGA	-0.39 (0.07)	<0.001	-0.32 (0.09)	<0.001

All linear regression models include the 12-month PCS or MCS as the response variable and age, gender, disease duration and treatment as covariates.
